# The impact of maternal anti-inflammatory drugs on surgical anesthesia-induced neuroinflammation and cognitive impairment in offspring mice

**DOI:** 10.3389/fncel.2024.1481630

**Published:** 2024-10-08

**Authors:** Dongdong Chai, Hong Jiang, Hua Liu

**Affiliations:** Department of Anesthesiology and Critical Care Medicine, Shanghai Ninth People’s Hospital Affiliated to Shanghai Jiao Tong University School of Medicine, Shanghai, China

**Keywords:** neuroinflammation, cognitive dysfunction, tau proteins, sevoflurane, ibuprofen

## Abstract

**Background:**

The impact of maternal surgery combined with general anesthesia on neuroinflammation and the development of learning and memory impairment in offspring remains unclear. This study utilized a pathogen-free laparotomy model to investigate these changes during the second trimester, as well as their response to anti-inflammatory therapy.

**Methods:**

C57BL/6 pregnant mice at the 14.5-day embryo stage (E 14.5) were either exposed to sevoflurane anesthesia alone or underwent laparotomy procedure. The neuroinflammatory response was evaluated at 7, 14, 21, and 28 days postnatal (P7, P14, P21, P28). Tau phosphorylation and cognitive ability were assessed at P28 and P30, respectively. The impact of perioperative administration of ibuprofen (60 mg/kg) on these aforementioned changes was subsequently evaluated.

**Results:**

In the laparotomy group, levels of inflammatory factors (IL-4, IL-8, IL-17A, TGF-β, M-CSF, CCL2) in the brains of offspring mice, including the cerebral cortex and hippocampus, remained consistently elevated from P7 to P28. At P14, while the majority of inflammatory cytokine has no statistical difference, there was still a significant reactivation of inflammatory cytokines observed in the frontal cortex and hippocampus at P28. Furthermore, abnormal phosphorylation of tau and deficits in learning and memory were observed at P28 and P30. Administration of perioperative ibuprofen led to improvements in cognitive performance, reduction of systemic inflammation, and inhibiting abnormal phosphorylation of tau in the frontal cortex and hippocampus.

**Conclusion:**

Our findings indicate that cognitive dysfunction is correlated with elevated levels of inflammatory cytokines and tau phosphorylation. Cognitive impairment and tau phosphorylation after laparotomy can persist at least until P28. Anti-inflammatory medications have been shown to enhance cognitive function by rapidly reducing inflammation in the brain, while also impacting neurological changes. This discovery may have implications for the development of treatment strategies aimed at managing cognitive impairment in post-operative patients.

## 1 Introduction

Over the past decade, extensive research has been conducted to investigate the potential neurotoxicity associated with prolonged exposure to anesthetics (Cao et al., 2012; Lin and Zuo, 2011; Zuo, 2012). Animal and preclinical studies have increasingly provided evidence suggesting that general anesthetics can induce neuroinflammation in the developing brain, ultimately leading to long-term neurodevelopmental deficits (Shen et al., 2013; Shi et al., 2017; Zhao et al., 2020; Wu et al., 2019; Tian et al., 2018). It is estimated that approximately 1–2% of pregnant women may require surgical intervention for conditions such as ovarian cyst torsion, appendicitis, strangulated hernia, or trauma during pregnancy. The surgical risk in these cases is comparable to that of the general population. However, ensuring the safety of both the pregnant woman and the fetus during anesthesia has become a significant concern in the surgical process.

Given that the majority of non-obstetric and fetal interventional procedures during pregnancy occur in the mid-pregnancy stage (Zhao et al., 2011), it is crucial to prioritize the safety of both the pregnant woman and the fetus during anesthesia (Goodman, 2002). While research has indicated an increased risk of fetal cognitive impairment due to early and multiple exposures to general anesthetics during pregnancy, there has been limited attention given to the neurodevelopmental effects of general anesthesia on fetuses when administered to pregnant women during this stage. In order to replicate these clinical scenarios, we performed surgical procedures on animals while they were under general anesthesia. Our primary objective was to investigate whether undergoing a surgical procedure while under anesthesia would exacerbate the detrimental effects on the brain that are observed in clinical settings.

Prior research has presented evidence indicating that the primary neurotoxic effects of sevoflurane involve the accumulation of β-amyloid protein (Aβ) (Zhang B. et al., 2013), neuroinflammation (Zhang L. et al., 2013), and a decrease in synaptic plasticity (Haseneder et al., 2009). Furthermore, sevoflurane has been demonstrated to induce the expression of inflammatory factors such as TNF-α and IL-1β. This induction leads to neuroinflammation, neuronal damage, and ultimately contributes to long-term cognitive dysfunction in adulthood or postoperative cognitive dysfunction (Rosczyk et al., 2008; Vacas et al., 2013). Moreover, it is widely acknowledged that inflammatory mechanisms are thought to play a significant role in the pathophysiology of immunological responses in certain neurodegenerative diseases, such as Alzheimer’s disease. This study design aligns with the methodology of numerous clinical studies comparing a surgical group receiving anesthesia with an anesthesia-only group (Moller et al., 1998; Monk et al., 2008; Newman et al., 2001). Previous animal experimental models commonly utilized bacterial endotoxin lipopolysaccharides or live bacterial infections to induce systemic inflammation (Yeh et al., 2018; Hoogland et al., 2015). However, a limitation of utilizing lipopolysaccharides and pathogens is the constrained timeframe of several days for experimentation. Therefore, we have opted to employ a laparotomy experimental model in order to replicate the cognitive dysfunction observed in some patients following surgery. In this work, a single surgical procedure was carried out without inflicting any damage to the intestinal tissue, by opening the belly to expose the small intestine for gentle manipulation. The intestine was then moved back into the abdominal cavity to where it had originally been. During this laparotomy procedure, our main objective was to investigate the impact of laparotomy on neurocognitive function and the inflammatory response in offspring mice. Previous studies utilizing a similar laparotomy experimental model have assessed the post-surgical effects for a duration of up to 7 days (Wan et al., 2007; Wan et al., 2010; Pan et al., 2016). However, in this study, we extended the examination time frame from 7 days postnatal (P7) to 28 days postnatal (P28) in order to investigate a range of events, including cytokine gene expression, pathology development, and cognitive dysfunctions.

Our research sought to evaluate the effects of perioperative variables on neuroinflammation and the learning and memory deficits in offspring, specifically surgical operations performed under general anesthesia. Considering the frequent occurrence of general anesthesia and surgery as a combined approach in surgical patients, our aim was to provide insight into this topic.

## 2 Materials and methods

### 2.1 Animals

SLAC Laboratory Animal Co., Ltd., Shanghai, China, provided the pregnant C57BL/6 mice at embryonic day 14.5 (E 14.5). The Animal Care Committee of the Ninth People’s Hospital, Shanghai Jiao Tong University School of Medicine (Shanghai, China), examined and approved the study protocol. The mice were kept in a room with a temperature between 20 and 22°C and a humidity level of 50 ± 10% for a 12-h light-dark cycle. They were fed a regular food. Before being utilized in the experiment, they had a one-week acclimatization period and were allowed unlimited access to food and water. The light period was when all behavioral testing was carried out, particularly from 9 a.m. to 12 a.m.

### 2.2 Experimental protocols

A total of four groups—control (CON), sevoflurane anesthesia (SEV), laparotomy under sevoflurane anesthesia (LAP), and laparotomy with perioperative ibuprofen administration (LAP + Ibu) were randomly allocated to mice in our experiment. For each of the four sets of tests, the animals in the control group did not get anesthesia, did not have surgery, or received ibuprofen. Ibuprofen (Sigma-Aldrich, United States) was orally administered in drinking water to mid-pregnant mice at a dosage of 60 mg/kg/day (Heneka et al., 2005). Gavage was used for the first dosage 1 h prior to laparotomy, and the mice continued to drink ibuprofen solution in the second trimester until the pups were born. We measured mRNA expression and inflammatory cytokine concentration levels in the brain and hippocampus to investigate the biochemical features of laparotomy-induced inflammation. Phosphorylation of Tau protein and its associated signaling pathways were comparatively assessed on P28 using western blot analysis. Cognitive performance was assessed using a range of behavioral tests, including the Morris water maze and novel object recognition test.

### 2.3 Surgical and anesthetic procedures

The anesthesia was induced using 2.5% concentration of sevoflurane (Sevorane^™^, Abbott, Switzerland) and maintained at a constant level for 2 h using a rodent inhalation anesthesia apparatus (Harvard Apparatus, United States) with an oxygen flow rate of 0.8 L/min. The levels of anesthesia were monitored using a Datex^™^ infrared analyzer (Capnomac, Helsinki, Finland). Our surgical procedure was modified from previous studies (Zhang B. et al., 2013; Zhang L. et al., 2013; Haseneder et al., 2009). A longitudinal midline incision measuring 2.5 cm was performed in the abdomen during the surgical procedure, following exposure of the mouse to volatile anesthetics for a minimum of 3 min.

**Figure 1:**
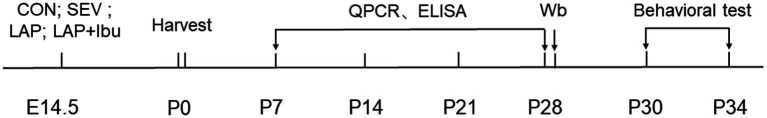
Flowchart of the experiment. CON, control group; SEV, sevoflurane alone; LAP, laparotomy under sevoflurane anesthesia; LAP + Ibu: laparotomy with ibuprofen administration.

Then, the intestine was everted approximately 10 cm and vigorously massaged for 30 s. The intestinal loop remained outside the abdominal cavity for one minute before reinsertion. A sterile intestinal suture (4-0, PS-2; Ethicon, United States) was used to suture the peritoneum lining and abdominal muscles as well as the skin in two layers. The wound was cleaned and closed with surgical sutures. The entire procedure was completed within 15 min, with continuous monitoring of respiratory rhythm and rate, as well as the color of the animal’s paw on the heating pad. Only mice in the anesthesia group received a consistent concentration and gas flow rate of 15 min of isoflurane anesthesia. Throughout the administration of anesthesia, rectal temperature was continuously monitored and maintained at 37°C with the use of a servo-controlled warming blanket (TCAT-2LV, Physitemp Instruments, Clifton, NJ). The mouse’s heart rate and pulse oxygen saturation were continuously monitored using the MouseOX Murine Plus Oximeter System (Starr Life Sciences Corporation, Oakmont, PA). The mice were permitted to maintain spontaneous respiration throughout the procedure. At E18.5, pregnant mice in each group underwent a cesarean section to deliver their pups after being deeply anesthetized with 0.7% pentobarbital sodium (10 mL/kg) injected into the abdomen. The mice in question are referred to as P0 mice on this day. The cerebral cortex and hippocampus tissues were collected at various time points, immediately frozen in liquid nitrogen, and stored at −80°C. The detailed experimental procedure is illustrated in Figure 1.

### 2.4 Real-time PCR and RNA extraction

Mice were carbon dioxide asphyxiated in a humane manner, following the American Veterinary Medical Association’s norms. Tissues were treated with the RNeasy Mini Kit (QIAGEN Hamburg, Germany) to extract total RNA. For analysis, we only employed isolated RNA samples with OD260/280 ratios >1.8 and OD260/230 ratios <2.0. Two milligrams of RNA were reverse-transcribed into 40 ng of complementary template DNA after further purification with the Ambion^®^ DNA-free^™^ DNA removal kit (Invitrogen, United States) and reverse transcription with the PrimeScript^™^ Master Mix kit (TAKARA, Japan). The SYBR^®^ Premix Ex Taq^™^ II kit (TAKARA, Japan) and the StepOnePlus^™^ real-time PCR system (Applied Biosystems, United States) were used for the PCR process. The amplification cycle reaction was run 40 times with the following parameters: 5 s of denaturation at 95°C and 30 s of annealing at 60°C. Through the use of 2^−ΔΔCt^, cytokine levels were standardized relative to the endogenous reference glyceraldehyde-3-phosphate dehydrogenase (GAPDH). The QuantStudio 6 Flex program was used to analyze the data. Table 1 presents the primer sequences for mice.

**Table 1:** The primer sequences of genes in this experiment.

Target genes	Forward primer	Reverse primer
IL-4	CGGCAACTTTGTCCACGGA	TCTGTTACGGTCAACTCGGTG
IL-8	TGCCGTGACCTCAAGATGTGCC	CATCCACAAGCGTGCTGTAGGTG
IL-17A	GAGCTTCATCTGTGTCTCTGAT	GCCAAGGGAGTTAAAGACTTTG
TGF-β	GGCACCATCCATGACATGAACCG	GCCGTACACAGCAGTTCTTCTCTG
M-CSF	AGCTTTACGAGAGCTCTTTTGC	CACATCCTCCTCAGGACCTT
CCL2	ATTCAACGGCACAGTCAA	CTCGCTCCTGGAAGATGG
GAPDH	ATTCAACGGCACAGTCAA	CTCGCTCCTGGAAGATGG

### 2.5 Western blot and SDS-PAGE analysis

In accordance with the American Veterinary Medical Association’s recommendations, mice were humanely put to death via carbon dioxide asphyxiation. Tissues from the frontal brain and hippocampus were cut apart for protein blotting. On ice, tissues were homogenized in RIPA buffer using a combination of protease inhibitors. After that, the homogenates were centrifuged for 30 min at 4°C at 13,000 g. The protein concentration was then assessed using the Bradford assay after the resultant supernatant was saved. The Pierce BCA Protein Assay Kit (Beyotime Institute of Biotechnology) was used to quantify proteins. Following the procedure previously outlined (Chai et al., 2020), total proteins were collected and electrophoresed on a 10% polyacrylamide gel. The membranes were sealed with 5% skim milk powder and then incubated with particular primary antibodies for an entire night at 4°C. Then, employing secondary antibodies linked to horseradish peroxidase (DAKO, Denmark), the experiments were carried out. Chemiluminescence (ECL or ECL plus, Amersham GE Healthcare, United Kingdom) was then used to depict the intensity of the immunoreactive band signal. By loading the GAPDH antibody onto the gel, all immunoblots were normalized using this internal reference. Primary antibodies from Thermo Fisher Scientific, phosphorylated tau (Ser404) from Santa Cruz Biotechnology, phosphorylated Jak2, Stat3, GSK3β, phosphorylated GSK3β, MAPK, phosphorylated MAPK, and SAPK, phosphorylated SAPK, were among those utilized. Image-J software was used to quantify the chemiluminescent bands’ intensity. The relative expression level of the protein was determined by dividing the gray value of the target band by that of the reference band, with GAPDH serving as the internal reference protein.

### 2.6 Enzyme-linked immunosorbent assay

Weighing frozen mouse brain samples and homogenizing them in 5% Tris-buffered saline containing a combination of protease inhibitors was the process of preparing brain tissue for biochemical examination. Aliquots of homogenates were kept at −80°C for subsequent biochemical analysis. Blind evaluations were performed on all biochemical markers. Enzyme-linked immunosorbent assay (ELISA) kits were used to ascertain the concentrations of IL-4, IL-8, IL-17A, TGF-β, M-CSF, and CCL2 in the frontal cortex and hippocampus, following the guidelines provided by the manufacturer (Abcam, United Kingdom). In short, 96-well plates coated with particular antibodies were filled with supernatants from either hippocampal or frontal cortex tissue. Using an enzyme marker (Thermo Fisher Scientific, United States), the absorbance values of the samples were determined at 450 nm following the reaction of the enzyme and substrate.

### 2.7 Morris water maze

Morris water maze training and exploration tests were used to assess mice’s capacity for spatial learning and memory. A circular, white, 45-centimeter-deep pool with a diameter of 94 cm was used for the Morris water maze test. A non-toxic white water-based tempera paint is applied, and the pool is filled with 30 centimeters of opaque water. The temperature of the pool was kept constant at 22 ± 1°C. It was erroneously split into four conceptual sections. A 20-centimeter-diameter platform was sunk 1 cm below the water’s surface at the northwest quadrant’s center. The platform was taken down to conduct testing. During training and testing, four more maze clues were set around the pool and maintained in the same locations: traffic cones, colored posters, and two black and white construction paper drawings. A camera positioned above the pool’s center captured the swimming route, and a video tracking motion analysis system (Ethovision, Noldus, version 4.1) tracked movement. The light of 75 Lux filled the Morris water maze, which was encircled by white curtains that held hints that were hidden. For 4 days, the mice were taught using the Morris water maze four times a day.

The animals were released into one of four predetermined quadrants at the end of each training session. For 60 s, or until they arrived at the escape platform, they were free to swim. The animals were taken out of the water after they arrived at the platform and stood there for 15 s. In order to give spatial information regarding the platform’s position, during training, if the animals could not locate it in 60 s, they were manually directed and left on the platform for 15 s. The platform stayed in the same quadrant throughout every test. The mice were taken out of the pool and carefully patted dry with a towel before being put in a heated cage beneath a typical shoebox cage that had a low heat pad. They were returned to the cage after at least 5 min. The experiment was run on the fifth day. The animal was put in the opposite quadrant of its prior location and the platform was taken out of the water for the test. The animals were given 60 s to swim during the test. Record the number of times the mouse cross over the previous station position, the time it takes to get there (escape latency), the amount of time the mouse spend in the target quadrant (seconds, s), and the pace at which the mouse are swimming. Every behavior analysis and test was conducted in blind.

### 2.8 Novel object recognition test

On postnatal day 30 (P30), the animals were given the novel object recognition (NOR) task to assess their ability to recognize a novel object in a controlled setting. The mice were first housed for 24 h in an empty open field arena before spending 10 min in an arena containing two identical sample objects (A + A). Following a 24-h period of confinement, the mice were once again placed in the same arena with two objects: one new (A + B) and the other identical to the sample. The test objects were a paper box filled with sand that was covered in different colored waterproof tape and a Falcon tissue culture flask that was 9.5 cm in height, 2.5 cm in depth, and 5.5 cm in width. The two items had the same dimensions. The discrimination index, which is computed as the ratio of exploration time between the two objects, was used to evaluate recognition memory. The objects were placed in opposing and symmetrical corners of the arena for both the testing and familiarization phases. Within each group of tests, the distribution of unfamiliar versus familiar objects was counterbalanced. Blinding was used for all behavioral assessments and analysis.

### 2.9 Statistical analysis

GraphPad Prism (version 6.0, Graph Pad Software Inc., United States) was used for statistical analysis, and the results are shown as the mean ± SEM. One-way ANOVA was used to examine the data, which included behavioral assessments, normalized band intensities in western blots, and relative mRNA levels of cytokines. The Tukey *post hoc* test was then used to assess the results. The Shapiro–Wilk, Kolmogorov–Smirnov, and D’Agostino-Pearson omnibus normality tests were used to determine the homogeneity of group variances and the normality of the data, respectively. *p* less than 0.05 was used to define statistical significance.

## 3 Results

### 3.1 Ibuprofen ameliorates neuroinflammation induced by laparotomy in the central nervous system of mice

**Figure 2:**
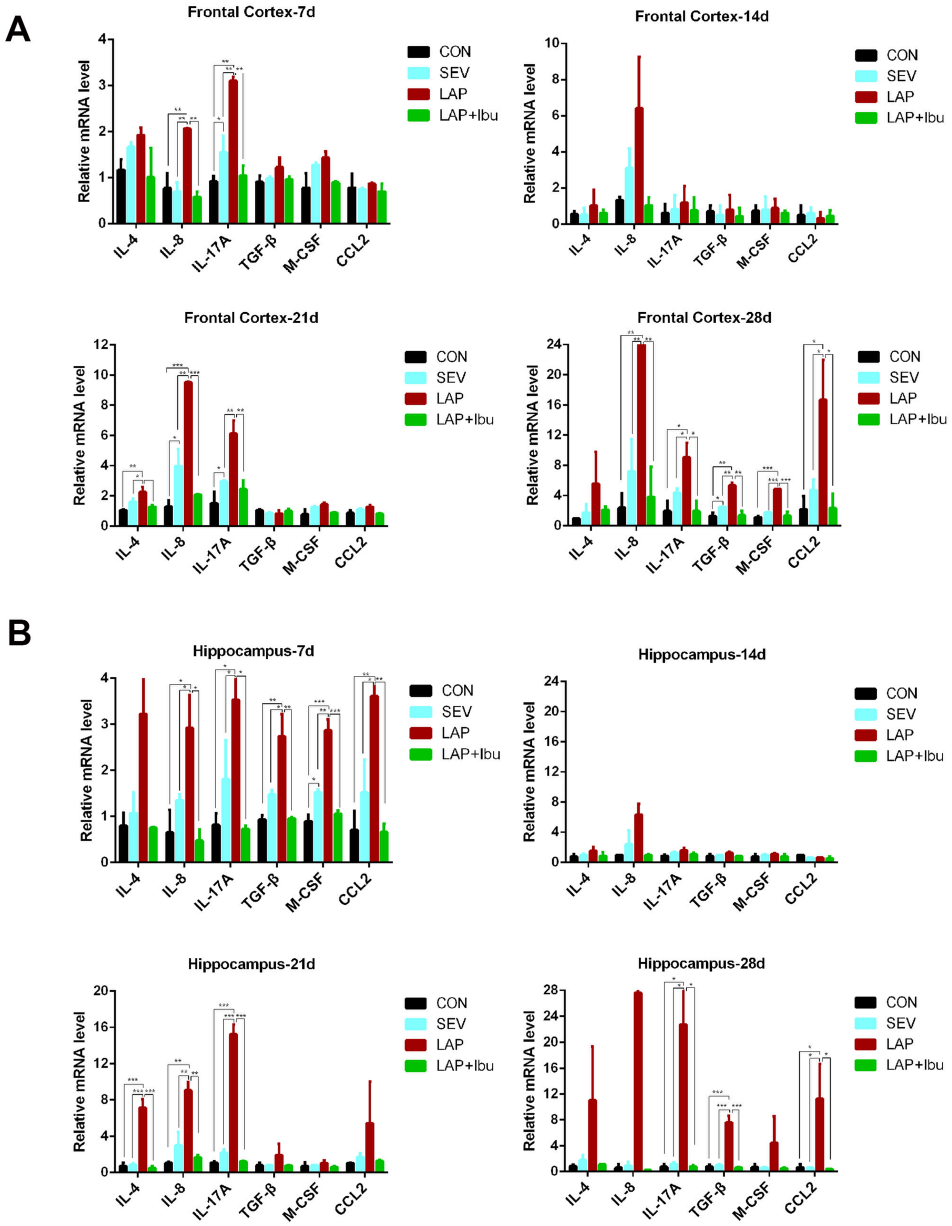
Laparotomy induced persistent neuroinflammation at different neurodevelopment stage, while ibuprofen improves laparotomy-induced neuroinflammation in mouse central nervous system. **(A)** Relative mRNA levels of inflammatory cytokines in the frontal cortex at various time points during the postoperative period. **(B)** Relative mRNA levels of inflammatory cytokines in the hippocampus at various time points following the postoperative period. *n* = 6–8, ^*^p < 0.05, ^**^p < 0.01, and ^***^p < 0.001.

No animals perished during the experiments. Data from all animals involved in the study were included for analysis. Regarding the expression levels of inflammatory factor mRNA, our findings suggest that sevoflurane alone did not induce significant changes in inflammatory factors compared to the control group. However, a significant increase in inflammatory factors was observed during sevoflurane-based surgery. Compared to the SEV group, the LAP group demonstrated evidence of neuroinflammation characterized by elevated levels of mRNA for pro-inflammatory cytokines interleukin-8 (IL-8) and IL-17A in both the frontal cortex (LAP vs. SEV, ^**^p = 0.002 and ^**^p = 0.002 respectively, Figure 2A) and the hippocampus (LAP vs. SEV, ^*^p = 0.026 and ^*^p = 0.042 respectively, Figure 2B) at P7. However, this phenomenon was not observed at P14. Interestingly, the LAP group exhibited a resurgence in elevated mRNA levels of IL-8 and IL-17A at both the cortex (LAP vs. SEV, ^**^p = 0.001 and ^**^p = 0.002 respectively, Figure 2A) and hippocampus (LAP vs. SEV, ^**^p = 0.002 and ^***^p < 0.001 respectively, Figure 2B) at P21, as well as in the cortex (LAP vs. SEV, ^**^p = 0.006 and ^*^p = 0.028 respectively, Figure 2A) at P28, compared to the SEV group. Furthermore, at P28, there was a rise in inflammatory factors such as TGF-β and CCL2 in both the frontal cortex (LAP vs. SEV, ^**^p = 0.002 and ^*^p = 0.017 respectively, Figure 2A) and hippocampus (LAP vs. SEV, ^***^p < 0.001 and ^*^p = 0.017 respectively, Figure 2B).

**Figure 3:**
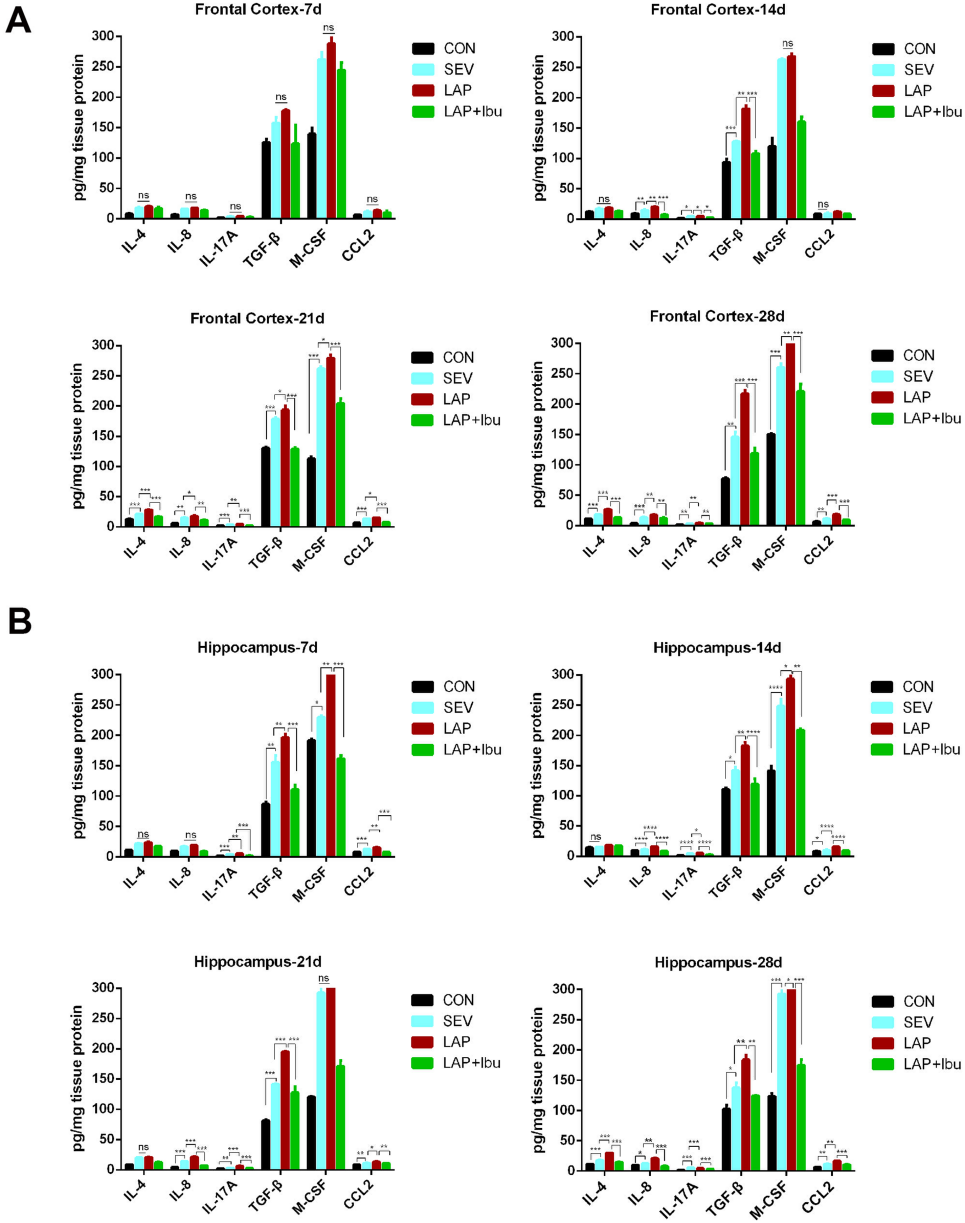
The cytokines concentration in the brain measured by MILLIPLEX assay under the different conditions. **(A)** The expression levels of IL-4, IL-8, IL-17A, TGF-β, M-CSF and CCL2 in the frontal cortex. **(B)** The expression levels of IL-4, IL-8, IL-17A, TGF-β, M-CSF and CCL2 in the hippocampus. *n* = 6–8, ^*^p < 0.05, ^**^p < 0.01, and ^***^p < 0.001.

Next, we proceeded with the measurements of cytokine protein expressions using the MILLIPLEX assay on whole tissue lysates obtained from P7–P28 for both the frontal cortex and hippocampus. In P7, compared to the SEV group, cytokines in the LAP group only showed an increase in hippocampal tissue (LAP vs. SEV, IL-17A: ^**^p = 0.001, TGF-β: ^**^p = 0.005, M-CSF: ^**^p = 0.001, CCL2: ^**^p = 0.004, Figure 3B), with no significant improvement observed in the cerebral cortex (Figure 3A). After P7, a substantial quantity of inflammatory factors was discharged. Compared to the SEV group, levels of circulating cytokines such as IL-8 and IL-17A remained elevated up to P28 in both the frontal cortex (LAP vs. SEV, ^**^p = 0.008 and ^**^p = 0.001 respectively, Figure 3A) and hippocampus (LAP vs. SEV, ^**^p = 0.001 and ^***^p < 0.001 respectively, Figure 3B) in the LAP group. Furthermore, the levels of TGF-β and CCL2 in the frontal cortex and hippocampus also demonstrated a consistent increase at various time points. Though the level of M-CSF also exhibited an increase, this heightened state was observed to be unstable at various time points and in different tissues. Perioperative ibuprofen consumption has been associated with a reduction in circulating cytokine levels, including IL-8 and IL-17A, as well as TGF-β, CCL2, and M-CSF (Figures 3A,B). The majority of the aforementioned inflammatory factors were significantly reduced in P21 (frontal cortex: LAP vs. LAP + Ibu, IL-8: ^**^p = 0.002, IL-17A: ^***^p < 0.001, TGF-β: ^***^p < 0.001, CCL2: ^***^p < 0.001; hippocampus: LAP vs. LAP + Ibu, IL-8: ^***^p < 0.001, IL-17A: ^***^p < 0.001, TGF-β: ^***^p < 0.001, CCL2: ^**^p = 0.002) and P28 (frontal cortex: LAP vs. LAP + Ibu, IL-8: ^**^p = 0.003, IL-17A: ^**^p = 0.004, TGF-β: ^***^p < 0.001, CCL2: ^***^p < 0.001; hippocampus: LAP vs. LAP + Ibu, IL-8: ^***^p < 0.001, IL-17A: ^***^p < 0.001, TGF-β: ^**^p = 0.001, CCL2: ^***^p < 0.001, Figures 3A,B) indicating that the drug possesses multiple anti-inflammatory effects.

### 3.2 Ibuprofen reduces laparotomy-induced tau phosphorylation in the central nervous system of mice

**Figure 4:**
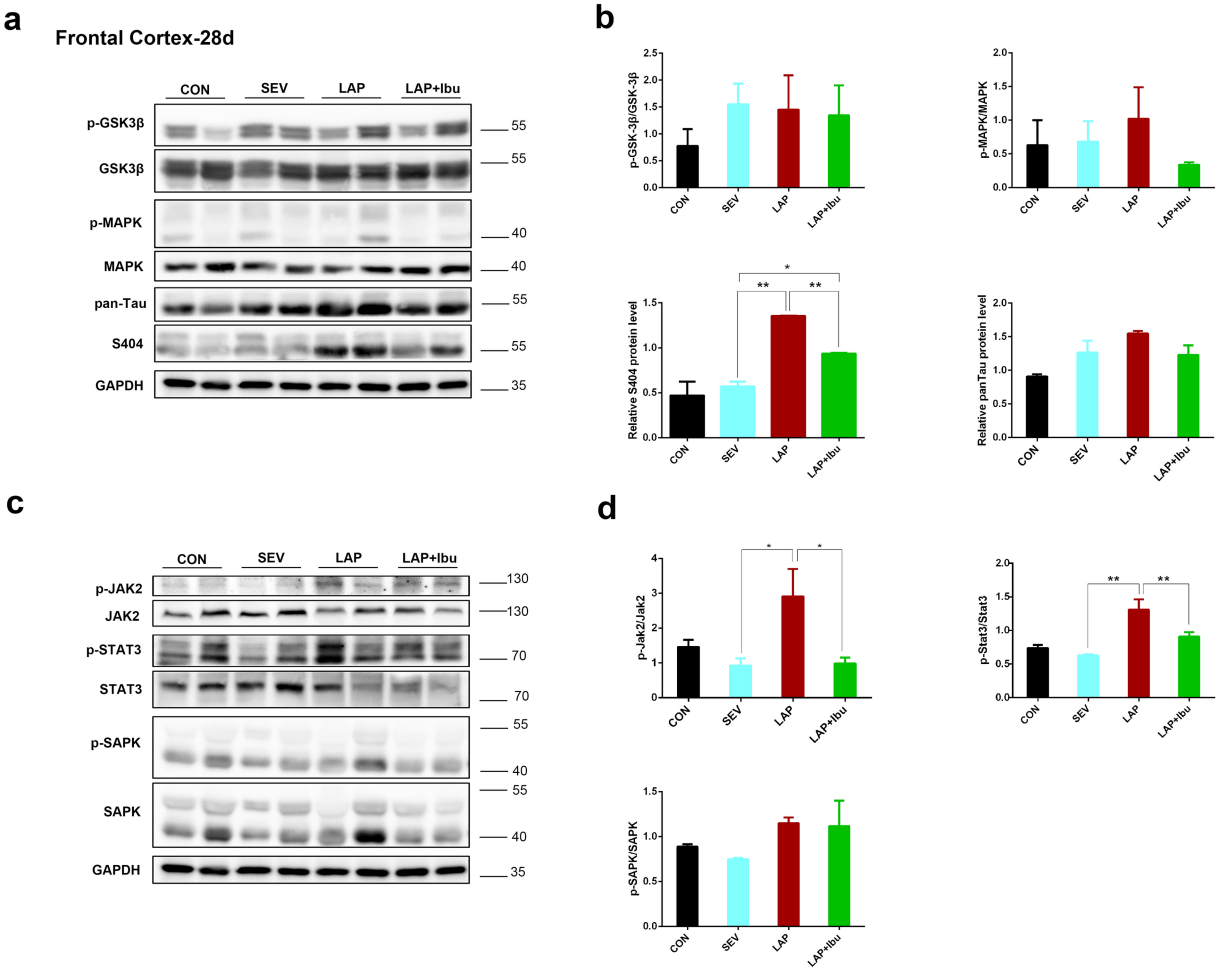
Phosphorylation of tau protein and related signaling pathways in the frontal cortex following sevoflurane anesthesia or laparotomy on P28. **(a)** Differential changes of GSK3β, MAPK and tau phosphorylation levels in the frontal cortex. **(b)** Quantification of the protein expression relative to GAPDH. **(c)** Relative levels of p-Jak2/Jak2, p-Stat3/Stat3, and p-SAPK/SAPK in the frontal cortex were evaluated by using western blot analysis. **(d)** Quantification of the protein expression relative to GAPDH. For each panel, *n* = 4, ^*^p < 0.05, ^**^p < 0.01, and ^***^p < 0.001.

**Figure 5:**
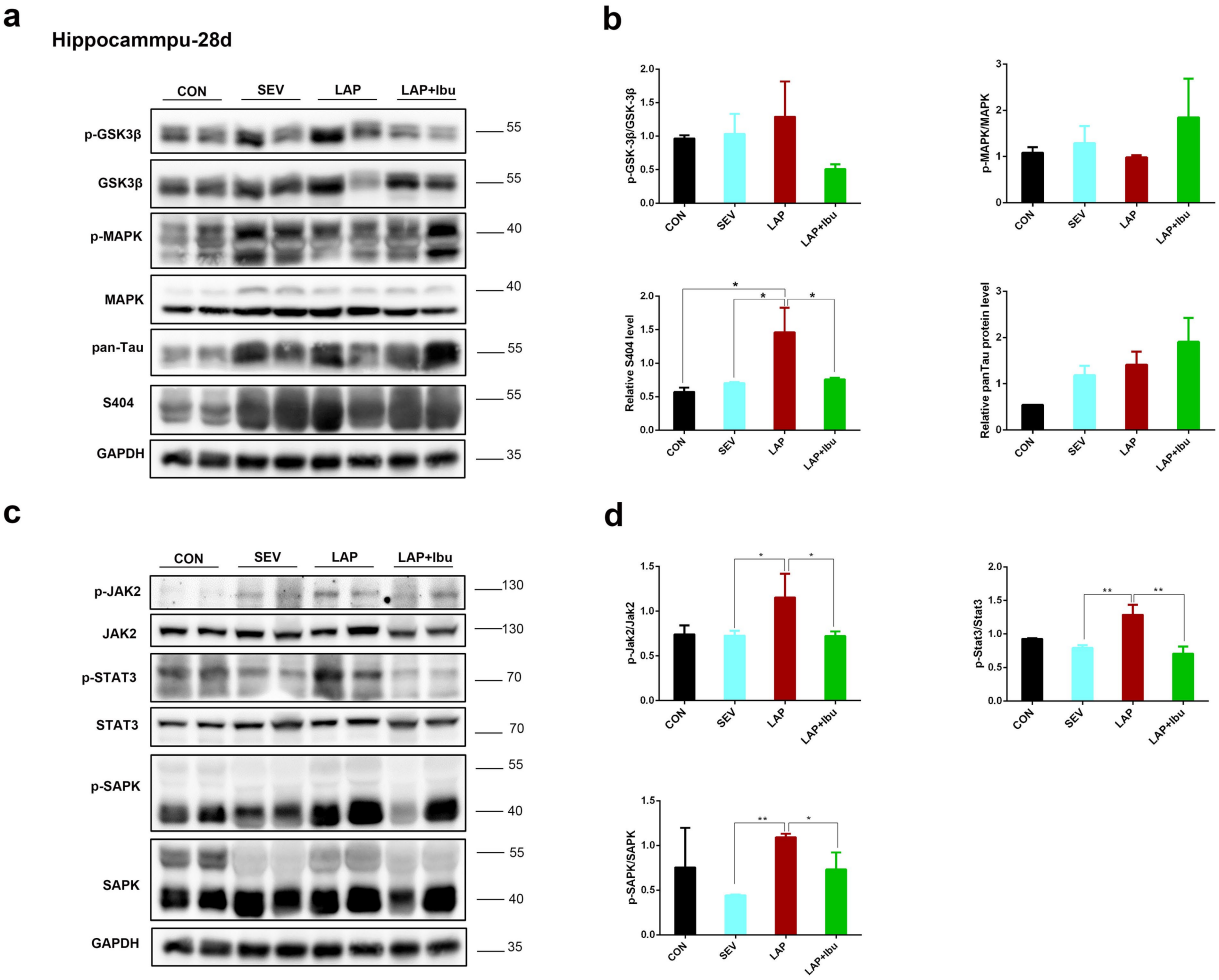
Phosphorylation of tau protein and related signaling pathways in the hippocampus following sevoflurane anesthesia or laparotomy on P28. **(a)** Differential changes of GSK3β, MAPK and tau phosphorylation levels in the hippocampus. **(b)** Quantification of the protein expression relative to GAPDH. **(c)** Relative levels of p-Jak2/Jak2, p-Stat3/Stat3, and p-SAPK/SAPK in the hippocampus were evaluated by using western blot analysis. **(d)** Quantification of the protein expression relative to GAPDH. For each panel, *n* = 4, ^*^p < 0.05, ^**^p < 0.01, and ^***^p < 0.001.

Numerous studies have clearly shown the predictive significance of aberrant tau protein phosphorylation in the etiology of postoperative altered cognitive impairment and neuronal death in human disorders like Alzheimer’s disease (AD) (Monk et al., 2008; Newman et al., 2001). The tau phosphorylation sites that were tested were selected based on previous literature. In line with the timing of mRNA and cytokine changes, we have opted to directly observe alterations in protein phosphorylation within the cerebral cortex and hippocampus of P28 mice. Compared to the SEV group, an increase in the expression of tau phosphorylation site S404 was observed in the frontal cortex (LAP vs. SEV, ^**^p = 0.001, Figures 4a,b) and hippocampus (LAP vs. SEV, ^*^p = 0.015, Figures 5a,b) following laparotomy on the P28. Phosphorylation of Tau depends on the balance between kinase and phosphatase activities. Compared to the SEV group, both JAK2 and STAT3 are up-regulated through phosphorylation in the frontal cortex (LAP vs. SEV, ^*^p = 0.03 and ^**^p = 0.003 respectively, Figures 4c,d) and the hippocampus (LAP vs. SEV, ^*^p = 0.044 and ^**^p = 0.006 respectively, Figures 5c,d) in the LAP group, indicating the persistent presence of cellular stress.

The findings also indicated that the levels of Jak2 and Stat3 did not increase when sevoflurane was administered alone, however, there was a significant increase in protein changes observed when laparotomy was performed in combination with sevoflurane. Similarly, reductions in tau protein phosphorylation (S404) were also observed in the frontal cortex (LAP vs. LAP + Ibu, ^**^p = 0.007, Figure 4b) and the hippocampus (LAP vs. LAP + Ibu, ^*^p = 0.02, Figure 5b) of the mice that were administered ibuprofen. The activity of the stress-related signaling pathway JAK and STAT3 kinase decreased after the administration of ibuprofen in the frontal cortex (LAP vs. LAP + Ibu, ^*^p = 0.03 and ^**^p = 0.003 respectively, Figure 4d) and the hippocampus (LAP vs. LAP + Ibu, ^*^p = 0.043 and ^**^p = 0.003 respectively, Figure 5d). This suggested that the stress signaling pathway may serve as the primary mechanism mediating the neuroprotective effect of ibuprofen on tau phosphorylation induced by open surgery. The significance of tau kinase GSK3β, as well as other cell survival kinases and tau phosphatases, was relatively minimal. We did not observe any correlation between the activities of GSK3β, SAPK, MAPK, and the phosphorylation status of the tau protein. Tau disease may emerge as a result of continued phosphorylation of tau protein brought on by persistent neuroimmune reactions. This neuroinflammation and phosphorylation of tau proteins may also contribute to cognitive dysfunctions, as demonstrated by the performance in the water morris maze and novel object recognition tests in offspring mice.

### 3.3 Ibuprofen alleviates laparotomy-induced cognitive impairment without impacting motor function

**Figure 6:**
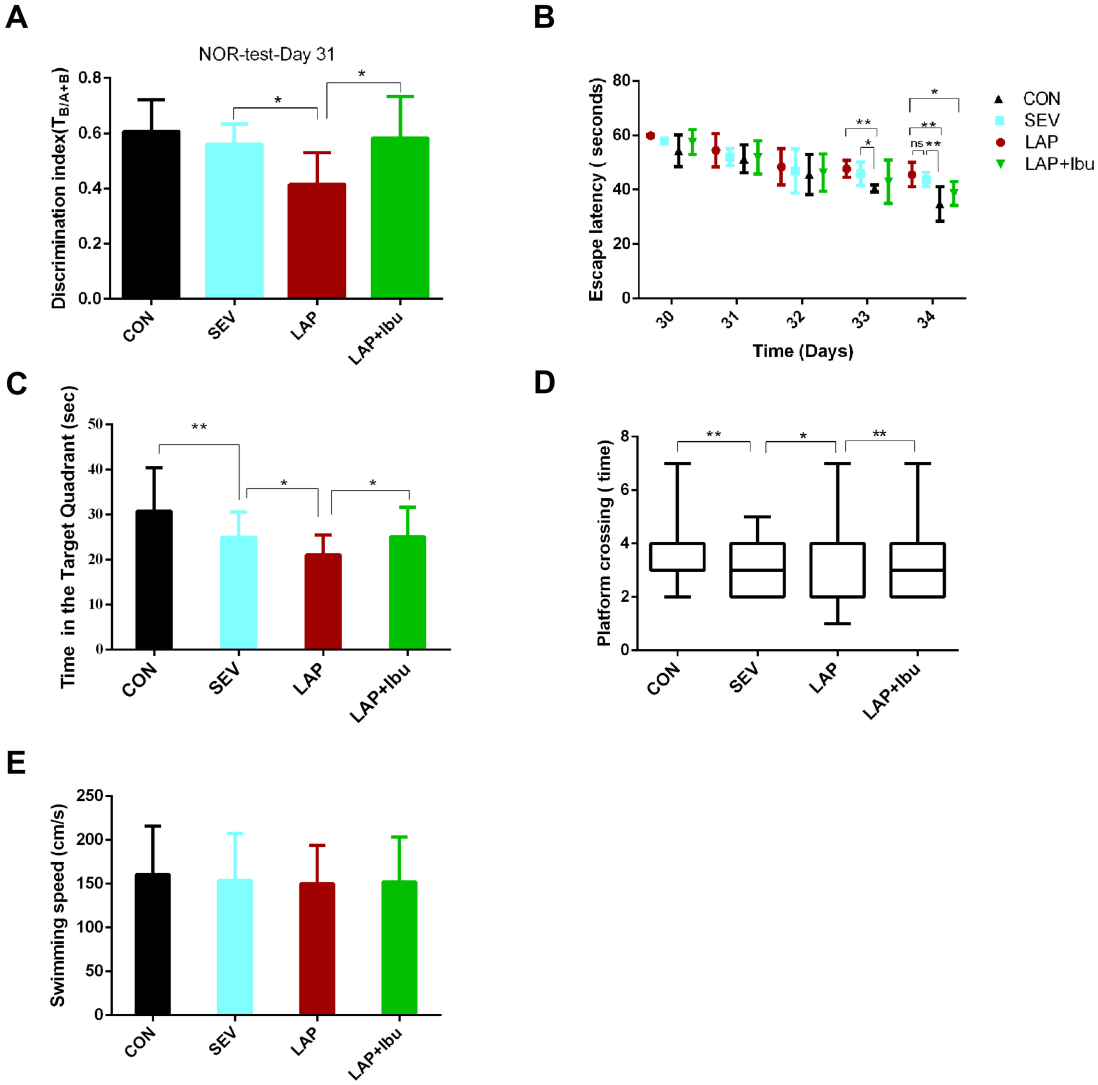
Persistent cognitive impairment during postoperative period, while perioperative ibuprofen intervention prevented cognitive dysfunction. **(A)** In the novel object recognition (NOR) test, LAP decreased the discrimination index (DI), while reduction in decline of DI in the NOR test was observed following laparotomy with ibuprofen treatment (LAP vs. SEV, ^*^p = 0.035, LAP vs. LAP + Ibu, ^*^p = 0.02, *n* = 8). **(B)** The LAP group exhibited an increased escape latency on the test day in comparison to the CON group, whereas a reduction in escape latency was noted following treatment with ibuprofen (LAP vs. CON, ^**^p = 0.003, LAP vs. LAP + Ibu, ^*^p = 0.046). **(C)** LAP decreased the time spent in the target quadrant, while increased the time in the target quadrant following treatment with ibuprofen (LAP vs. SEV, ^*^p = 0.035, LAP vs. LAP + Ibu, ^*^p = 0.013). **(D)** LAP decreased the platform crossing times, while a reduction in crossing times decline in the MWM test after laparotomy with ibuprofen treatment was observed (LAP vs. SEV, ^*^p = 0.019, LAP vs. LAP + Ibu, ^**^p = 0.002). **(E)** LAP did not affect swimming speed compared with the same variables in the SEV group mice. B–E: *n* = 8–11, ^*^p < 0.05, ^**^p < 0.01, and ^***^p < 0.001.

Compared to the SEV group, the LAP group demonstrated a significant impairment in recognition memory, as evidenced by a lower discrimination index on the New Object Recognition (NOR) test (LAP vs. SEV, ^*^p = 0.035, Figure 6A). Furthermore, spatial memory was evaluated using the Morris water maze test from P30 to P34. The LAP group exhibited a shorter duration in the target quadrant (LAP vs. SEV, ^*^p = 0.0355, Figure 6C) and a lower number of platform crossings (LAP vs. SEV, ^*^p = 0.019, Figure 6D) compared to the SEV group.

On the day of testing, there was no significant difference in escape latency between the LAP and SEV groups (LAP vs. SEV, *p* > 0.05, Figure 6B), the escape latency of the LAP group was significantly longer than that of the CON group (LAP vs. CON, ^**^p = 0.003, Figure 6B), indicating an exacerbation of learning and memory impairment following laparotomy operation. These findings indicated that memory impairment persists from postnatal onset into adolescence, which is consistent with previous data. Ibuprofen alleviated these impairments (Figures 6B–D) without impacting motor activity, swimming speeds were consistent across all groups (Figure 6E).

## 4 Discussion

For a considerable period, there has been apprehension surrounding the capacity of anesthetics to trigger neurological irregularities. Preclinical studies have the potential to offer valuable mechanistic insights into the neurotoxicity associated with anesthesia. According to recent clinical research, a single, brief experience with general anesthesia in infancy may not always result in neurodevelopmental impairments (McCann et al., 2019; Sun et al., 2016). However, exposure of mid-pregnant mice to anesthetics has been demonstrated to have a detrimental impact on brain development, and the long-term cognitive impact of mid-pregnancy surgery under anesthesia on offspring remains uncertain.

In this study, we employed a clinically relevant surgical experimental model, specifically second-trimester surgery under sevoflurane anesthesia, to investigate the potential impact of such clinical procedures on the neural development of offspring mice. While previous experimental studies have explored the impacts of the second trimester, only a few have combined anesthesia and surgery into a novel model and evaluated the long-term neurodevelopmental changes in offspring mice. Our research findings suggest that performing laparotomy under anesthesia during the second trimester can trigger persistent inflammatory responses in the brains of offspring mice for at least one-month least after birth, leading to long-term impairments in learning and memory. However, we have found that these impairments can be alleviated through anti-inflammatory therapy.

Our experimental model demonstrated that laparotomy, as opposed to sevoflurane alone, resulted in heightened levels of inflammatory factors and tau phosphorylation. Compared to the SEV group, the LAP group showed continuous elevation of IL-8 and IL-17A from P7 to P28, along with an increase in other inflammatory factors during the later period. Among them, there were no statistically significant differences in the elevation of numerous inflammatory factors on P14, we hypothesize that the brain may be initiating a self-healing process at this juncture, however, the levels of inflammatory factors continue to increase thereafter. These alterations in inflammatory factors ultimately result in long-term learning and memory impairment in mice, without affecting motor function. Moreover, ibuprofen’s anti-inflammatory therapy has demonstrated a reduction in tau phosphorylation and an improvement in cognition. However, the mechanisms by which surgery under sevoflurane anesthesia induces neuroinflammation remain unknown and warrant further exploration.

In terms of the mechanism through which these cytokines exert their inflammatory effects, we have formulated the following assumptions. The inflammatory response triggered by sevoflurane and/or injury disrupts the delicate physiological balance between immune and neural processes, activates various cellular mechanisms, and leads to neuroinflammation (Liu et al., 2023). Neuroinflammation is characterized by the activation of glial cells, including astrocytes, microglia, and oligodendrocytes, as well as the proliferation of immune cells (e.g., Th cells), resulting in the release of pro-inflammatory factors for a variety of reasons. Activated microglia are recognized as a central hub in the neuroinflammatory response within the central nervous system (Lambertsen et al., 2019). Upon activation by diverse stimuli, microglia can transition from a resting state to a pro-inflammatory M1 phenotype. This transformation is accompanied by the release of numerous pro-inflammatory cytokines (such as interleukins and tumor necrosis factor-α) and chemokines including CCL2 (Cherry et al., 2020) and M-CSF (Wang et al., 2022). In response to pathological environments, astrocytes may express interleukin-1 (IL-1) and IL-8, while Th-17 cells exhibit an increased secretion of IL-17A (Zheng et al., 2024). The primary effector molecules secreted by Th2 cells include IL-4 (Altin et al., 2012). The overexpression of these pro-inflammatory cytokines and chemokines can lead to impaired learning and memory, diminished neural plasticity, and reduced neurogenesis. The results suggest that IL-8 and IL-17A show distinct changes in the cortex or hippocampus at different time points, indicating that these two inflammatory factors may play a significant role in the neurocognitive impairment caused by laparotomy. Pro-inflammatory cytokines, such as interleukins, particularly interleukin 8 and interleukin 17A, have been found to be elevated in patients with AD (Vaz et al., 2020). These cytokines act as chemokines in chronic neuroinflammation by facilitating the migration of immune cells, such as microglia, astrocytes, and other immune cells, towards inflamed areas. Additionally, they modulate the function of immune cells by binding to specific receptors (Mackay, 2001). Moreover, interleukin 17A has been demonstrated to also stimulate Aβ deposition, neuroinflammation, microglia activation, and an increase in neutrophil numbers (Mohammadi Shahrokhi et al., 2018). CCL2 is widely regarded as one of the most potent chemokines for microglia/macrophages. Previous research indicates that CCL2 may attract immune cells from both the peripheral and central nervous systems to the site of injury, leading to the initiation of a prolonged and harmful inflammatory response. It is probable that CCL2 solely contributes to cell recruitment, while the glial inflammatory response is mediated by secondary factors such as proximity to damaged neurons, pTau, or Aβ. An imbalance of M-CSF may lead to chronic inflammation and brain inflammation (Spath et al., 2017; Lotfi et al., 2019). Excessive production of M-CSF may induce reactive oxygen species (ROS) through brain infiltration and phagocytosis, leading to the development of spontaneous brain inflammation and neurological disorders (Spath et al., 2017). This study has determined that M-CSF did not exhibit persistent changes, indicating that this inflammatory alteration may be restored to normal levels through subsequent self-regulation. Transforming growth factor-β (TGF-β) is a potent immunosuppressive cytokine that can be expressed by nearly all cells throughout the body. Numerous studies have suggested that an elevation in TGF-β can potentially initiate an activated intracellular mitotic signaling cascade. Mitotic signals, which involve the activation of mitogen-activated protein kinases (MAPKs) and other protein kinases, have the potential to modify the phosphorylation state of structural proteins such as tau, ultimately leading to excessive phosphorylation deposition. Our experimental results also suggest that TGF-β levels may continue to increase until P28, potentially leading to excessive phosphorylation of the hippocampus in offspring during this time period.

It is widely acknowledged that inflammatory processes may contribute to the development of neurodegenerative changes, even prior to the occurrence of any tau alterations (Schuitemaker et al., 2009). The role of inflammation has been demonstrated in the development of postoperative cognitive dysfunction (Hovens et al., 2014; Qiu et al., 2016). The immediate increase in mRNA levels of pro-inflammatory cytokines in the frontal cortex and hippocampus at P7 indicates a significant neuroinflammation induced by laparotomy. Furthermore, these elevated levels persisted at P28, potentially as a result of the sustained release of pro-inflammatory cytokines IL-8, CCL2, IL-17A, and M-CSF, which may have contributed to the exacerbation of the severity of the inflammatory response.

An increasing amount of research indicates that aberrant tau hyper phosphorylation may be a major factor in the pathophysiology of anesthesia-induced neuronal death and cognitive decline (Le Freche et al., 2012) or peripheral surgery (Huang et al., 2018). However, the exact mechanism of action has not yet been precisely investigated. There is no doubt that systemic inflammation and neuroinflammation are significantly associated with cognitive dysfunction related to tau hyper phosphorylation. With the combined synergistic effects of the aforementioned factors, a significant increase in tau phosphorylation at P28 was observed in both the frontal cortex and hippocampus following surgery under anesthesia, as compared to anesthesia alone. In our study, a hallmark finding in many neurodegenerative disorders is the substantial increase in tau phosphorylation at S404 in the frontal cortex and hippocampus on P28. This increase characterizes the pathological profiles of cognitive impairment after laparotomy. The Tau protein has the potential to propagate and initiate a feedforward cycle that amplifies inflammation, even in cases where inflammation occurred prior to the formation of larger aggregates (Nilson et al., 2017). The fact that ibuprofen has a therapeutic effect further supports the idea that aberrant tau phosphorylation contributes to long-term learning and memory impairment.

It is well known that reducing the pro-inflammatory response helps to facilitate functional recovery from damage to the central nervous system. Furthermore, the suppression of systemic inflammation was found to prevent the observed changes in this study. The expression of kinases related to cell survival and tau phosphatase was comparatively subdued.

Our research has demonstrated that ibuprofen treatment avoids the cognitive effects linked to a decrease in tau phosphorylation after laparotomy (Lim et al., 2000). While we have demonstrated the benefits of administering the drug throughout the entire experimental period, we have not yet investigated whether a shorter course to dampen the initial inflammatory response would yield similar results. This question is of clinical significance, particularly with regards to the prolonged use of nonsteroidal drugs, especially in the perioperative period, may present an unfavorable risk-benefit ratio, especially among elderly patients. In summary, our findings suggest that neuroinflammation following surgery may persist for an extended period and result in adverse changes in brain function. However, these effects can be alleviated through the use of anti-inflammatory treatment. However, it is challenging to differentiate the effects of anesthesia and surgery on cognition due to the infrequency of surgeries performed without anesthesia. Even in postoperative patients who have received regional anesthesia instead of general anesthesia, cognitive impairment still occurs clinically (Silbert et al., 2014). Recreating this experimental condition in animal models would pose significant technical challenges.

## 5 Conclusion

In conclusion, we have developed a sensitive and stable animal paradigm to study neuropathological variations brought on by systemic inflammation. This model has comprehensively demonstrated several key components that contribute to the development of cognitive dysfunction, including inflammation and tau phosphorylation. Moreover, additional clarification of the brain and systemic inflammatory profiles during the stages of cognitive decline is necessary.

## Data Availability

The raw data supporting the conclusions of this article will be made available by the authors, without undue reservation.

## References

[ref1] Altin J. A. Goodnow C. C. Cook M. C. (2012). IL-10^+^ CTLA-4^+^ Th2 inhibitory cells form in a Foxp3-independent, IL-2-dependent manner from Th2 effectors during chronic inflammation. J. Immunol. 188, 5478–5488. doi: 10.4049/jimmunol.1102994, PMID: 22547705

[ref2] Cao L. Li L. Lin D. Zuo Z. (2012). Isoflurane induces learning impairment that is mediated by interleukin 1β in rodents. PLoS One 7:e51431. doi: 10.1371/journal.pone.0051431, PMID: 23251531 PMC3520904

[ref3] Chai D. Yan J. Li C. Sun Y. Jiang H. (2020). Sevoflurane inhibits neuronal migration and axon growth in the developing mouse cerebral cortex. Aging 12, 6436–6455. doi: 10.18632/aging.103041, PMID: 32271715 PMC7185136

[ref4] Cherry J. D. Meng G. Daley S. Xia W. Svirsky S. Alvarez V. E.. (2020). CCL2 is associated with microglia and macrophage recruitment in chronic traumatic encephalopathy. J. Neuroinflammation 17:370. doi: 10.1186/s12974-020-02036-4 33278887 PMC7718711

[ref5] Goodman S. (2002). Anesthesia for nonobstetric surgery in the pregnant patient. Semin. Perinatol. 26, 136–145. doi: 10.1053/sper.2002.32203 12005471

[ref6] Haseneder R. Kratzer S. von Meyer L. Eder M. Kochs E. Rammes G. (2009). Isoflurane and sevoflurane dose-dependently impair hippocampal long-term potentiation. Eur. J. Pharmacol. 623, 47–51. doi: 10.1016/j.ejphar.2009.09.022, PMID: 19765574

[ref7] Heneka M. T. Sastre M. Dumitrescu-Ozimek L. Hanke A. Dewachter I. Kuiperi C.. (2005). Acute treatment with the PPARgamma agonist pioglitazone and ibuprofen reduces glial inflammation and Aβ1-42 levels in APPV717I transgenic mice. Brain 128, 1442–1453. doi: 10.1093/brain/awh452, PMID: 15817521

[ref8] Hoogland I. C. Houbolt C. van Westerloo D. J. van Gool W. A. van de Beek D. (2015). Systemic inflammation and microglial activation: systematic review of animal experiments. J. Neuroinflammation 12:114. doi: 10.1186/s12974-015-0332-6, PMID: 26048578 PMC4470063

[ref9] Hovens I. B. Schoemaker R. G. van der Zee E. A. Absalom A. R. Heineman E. van Leeuwen B. L. (2014). Postoperative cognitive dysfunction: involvement of neuroinflammation and neuronal functioning. Brain Behav. Immun. 38, 202–210. doi: 10.1016/j.bbi.2014.02.002 24517920

[ref10] Huang C. Irwin M. G. GTC W. RCC C. (2018). Evidence of the impact of systemic inflammation on neuroinflammation from a non-bacterial endotoxin animal mode. J. Neuroinflammation 15:147. doi: 10.1186/s12974-018-1163-z, PMID: 29776428 PMC5960121

[ref11] Lambertsen K. L. Finsen B. Clausen B. H. (2019). Post-stroke inflammation-target or tool for therapy? Acta Neuropathol. 137, 693–714. doi: 10.1007/s00401-018-1930-z, PMID: 30483945 PMC6482288

[ref12] Le Freche H. Brouillette J. Fernandez-Gomez F. J. Patin P. Caillierez R. Zommer N.. Tau phosphorylation and sevoflurane anesthesia: an association to postoperative cognitive impairment. Anesthesiology 116, 779–787. doi: 10.1097/ALN.0b013e31824be8c7 22343471

[ref13] Lim G. P. Yang F. Chu T. Chen P. Beech W. Teter B.. (2000). Ibuprofen suppresses plaque pathology and inflammation in a mouse model for Alzheimer’s disease. J. Neurosci. 20, 5709–5714. doi: 10.1523/JNEUROSCI.20-15-05709.2000, PMID: 10908610 PMC6772529

[ref14] Lin D. Zuo Z. (2011). Isoflurane induces hippocampal cell injury and cognitive impairments in adult rats. Neuropharmacology 61, 1354–1359. doi: 10.1016/j.neuropharm.2011.08.011, PMID: 21864548 PMC3189329

[ref15] Liu Y. Yang W. Xue J. Chen J. Liu S. Zhang S.. (2023). Neuroinflammation: the central enabler of postoperative cognitive dysfunction. Biomed. Pharmacother. 167:115582. doi: 10.1016/j.biopha.2023.115582, PMID: 37748409

[ref16] Lotfi N. Thome R. Rezaei N. Zhang G. X. Rezaei A. Rostami A.. (2019). Roles of GM-CSF in the pathogenesis of autoimmune diseases: an update. Front. Immunol. 10:1265. doi: 10.3389/fimmu.2019.01265, PMID: 31275302 PMC6593264

[ref17] Mackay C. R. (2001). Chemokines: immunology’s high impact factors. Nat. Immunol. 2, 95–101. doi: 10.1038/84298 11175800

[ref18] McCann M. E. de Graaff J. C. Dorris L. Disma N. Withington D. Bell G.. (2019). Neurodevelopmental outcome at 5 years of age after general anaesthesia or awake-regional anaesthesia in infancy (GAS): an international, multicentre, randomised, controlled equivalence trial. Lancet 393, 664–677. doi: 10.1016/S0140-6736(18)32485-1, PMID: 30782342 PMC6500739

[ref19] Mohammadi Shahrokhi V. Ravari A. Mirzaei T. Zare-Bidaki M. Asadikaram G. Arababadi M. K. (2018). IL-17A and IL-23: plausible risk factors to induce age-associated inflammation in Alzheimer’s disease. Immunol. Investig. 47, 812–822. doi: 10.1080/08820139.2018.1504300 30081688

[ref20] Moller J. T. Cluitmans P. Rasmussen L. S. Houx P. Rasmussen H. Canet J.. (1998). Long-term postoperative cognitive dysfunction in the elderly ISPOCD1 study. ISPOCD investigators. International Study of Post-Operative Cognitive Dysfunction. Lancet 351, 857–861. doi: 10.1016/S0140-6736(97)07382-0, PMID: 9525362

[ref21] Monk T. G. Weldon B. C. Garvan C. W. Dede D. E. van der Aa M. T. Heilman K. M.. (2008). Predictors of cognitive dysfunction after major noncardiac surgery. Anesthesiology 108, 18–30. doi: 10.1097/01.anes.0000296071.19434.1e, PMID: 18156878

[ref22] Newman M. F. Kirchner J. L. Phillips-Bute B. Gaver V. Grocott H. Jones R. H.. (2001). Longitudinal assessment of neurocognitive function after coronary-artery bypass surgery. N. Engl. J. Med. 344, 395–402. doi: 10.1056/NEJM200102083440601, PMID: 11172175

[ref23] Nilson A. N. English K. C. Gerson J. E. Barton Whittle T. Nicolas Crain C. Xue J.. (2017). Tau oligomers associate with inflammation in the brain and retina of tauopathy mice and in neurodegenerative diseases. J. Alzheimers Dis. 55, 1083–1099. doi: 10.3233/JAD-160912, PMID: 27716675 PMC5147514

[ref24] Pan K. Li X. Chen Y. Zhu D. Li Y. Tao G.. (2016). Deferoxamine pre-treatment protects against postoperative cognitive dysfunction of aged rats by depressing microglial activation via ameliorating iron accumulation in hippocampus. Neuropharmacology 111, 180–194. doi: 10.1016/j.neuropharm.2016.09.004 27608977

[ref25] Qiu L. L. Ji M. H. Zhang H. Yang J. J. Sun X. R. Tang H.. (2016). NADPH oxidase 2-derived reactive oxygen species in the hippocampus might contribute to microglial activation in postoperative cognitive dysfunction in aged mice. Brain Behav. Immun. 51, 109–118. doi: 10.1016/j.bbi.2015.08.002, PMID: 26254234

[ref26] Rosczyk H. A. Sparkman N. L. Johnson R. W. (2008). Neuroinflammation and cognitive function in aged mice following minor surgery. Exp. Gerontol. 43, 840–846. doi: 10.1016/j.exger.2008.06.004, PMID: 18602982 PMC2756971

[ref27] Schuitemaker A. Dik M. G. Veerhuis R. Scheltens P. Schoonenboom N. S. M. Hack C. E.. (2009). Inflammatory markers in AD and MCI patients with different biomarker profiles. Neurobiol. Aging 30, 1885–1889. doi: 10.1016/j.neurobiolaging.2008.01.014, PMID: 18378357

[ref28] Shen X. Dong Y. Xu Z. Wang H. Miao C. Soriano S. G.. (2013). Selective anesthesia-induced neuroinflammation in developing mouse brain and cognitive impairment. Anesthesiology 118, 502–515. doi: 10.1097/ALN.0b013e3182834d77, PMID: 23314110 PMC3580002

[ref29] Shi Y. Wang G. Li J. Yu W. (2017). Hydrogen gas attenuates sevoflurane neurotoxicity through inhibiting nuclear factor kappa-light-chain-enhancer of activated B cells signaling and proinflammatory cytokine release in neonatal rats. Neuroreport 28, 1170–1175. doi: 10.1097/WNR.0000000000000899, PMID: 28926473

[ref30] Silbert B. S. Evered L. A. Scott D. A. (2014). Incidence of postoperative cognitive dysfunction after general or spinal anaesthesia for extracorporeal shock wave lithotripsy. Br. J. Anaesth. 113, 784–791. doi: 10.1093/bja/aeu163 24972789

[ref31] Spath S. Komuczki J. Hermann M. Pelczar P. Mair F. Schreiner B.. (2017). Dysregulation of the cytokine GM-CSF induces spontaneous phagocyte invasion and immunopathology in the central nervous system. Immunity 46, 245–260. doi: 10.1016/j.immuni.2017.01.007, PMID: 28228281

[ref32] Sun L. S. Li G. Miller T. L. Salorio C. Byrne M. W. Bellinger D. C.. (2016). Association between a single general anesthesia exposure before age 36 months and neurocognitive outcomes in later childhood. JAMA 315, 2312–2320. doi: 10.1001/jama.2016.6967, PMID: 27272582 PMC5316422

[ref33] Tian Y. Chen K. Y. Liu L. D. Dong Y. X. Zhao P. Guo S. B. (2018). Sevoflurane exacerbates cognitive impairment induced by Aβ1-40 in rats through initiating neurotoxicity, neuroinflammation, and neuronal apoptosis in rat hippocampus. Mediat. Inflamm. 2018, 1–10. doi: 10.1155/2018/3802324 PMC6198580 30402039

[ref34] Vacas S. Degos V. Feng X. Maze M. (2013). The neuroinflammatory response of postoperative cognitive decline. Br. Med. Bull. 106, 161–178. doi: 10.1093/bmb/ldt006, PMID: 23558082 PMC4990823

[ref35] Vaz M. Domingues C. Trindade D. Barra C. Oliveira J. M. Rosa I. M.. (2020). IL-8 and MCP-1 impact on tau phosphorylation and phosphatase. Curr. Alzheimer Res. 17, 985–1000. doi: 10.2174/1567205017666201130091129 33256579

[ref36] Wan Y. Xu J. Ma D. Zeng Y. Cibelli M. Maze M. (2007). Postoperative impairment of cognitive function in rats: a possible role for cytokine-mediated inflammation in the hippocampus. Anesthesiology 106, 436–443. doi: 10.1097/00000542-200703000-00007 17325501

[ref37] Wan Y. Xu J. Meng F. Bao Y. Ge Y. Lobo N.. (2010). Cognitive decline following major surgery is associated with gliosis, beta-amyloid accumulation, and tau phosphorylation in old mice. Crit. Care Med. 38, 2190–2198. doi: 10.1097/CCM.0b013e3181f17bcb, PMID: 20711073

[ref38] Wang T. Cui S. Hao L. Liu W. Wang L. Ju M.. (2022). Regulation of Th17/Treg balance by 27-hydroxycholesterol and 24S-hydroxycholesterol correlates with learning and memory ability in mice. Int. J. Mol. Sci. 23:4370. doi: 10.3390/ijms23084370, PMID: 35457188 PMC9028251

[ref39] Wu L. Zhao H. Weng H. Ma D. (2019). Lasting effects of general anesthetics on the brain in the young and elderly: “mixed picture” of neurotoxicity, neuroprotection and cognitive impairment. J. Anesth. 33, 321–335. doi: 10.1007/s00540-019-02623-7 30859366 PMC6443620

[ref40] Yeh C. H. Hsieh L. P. Lin M. C. Wei T. S. Lin H. C. Chang C. C.. (2018). Dexmedetomidine reduces lipopolysaccharide induced neuroinflammation, sickness behavior, and anhedonia. PLoS One 13:e0191070. doi: 10.1371/journal.pone.0191070, PMID: 29351316 PMC5774758

[ref41] Zhang B. Tian M. Zheng H. Zhen Y. Yue Y. Li T.. (2013). Effects of anesthetic isoflurane and desflurane on human cerebrospinal fluid Aβ and tau level. Anesthesiology 119, 52–60. doi: 10.1097/ALN.0b013e31828ce55d, PMID: 23438677 PMC3938174

[ref42] Zhang L. Zhang J. Yang L. Dong Y. Zhang Y. Xie Z. (2013). Isoflurane and sevoflurane increase interleukin-6 levels through the nuclear factor-kappa B pathway in neuroglioma cells. Br. J. Anaesth. 110, i82–i91. doi: 10.1093/bja/aet115, PMID: 23604542 PMC3667345

[ref43] Zhao S. Fan Z. Hu J. Zhu Y. Lin C. Shen T.. (2020). The differential effects of isoflurane and sevoflurane on neonatal mice. Sci. Rep. 10:19345. doi: 10.1038/s41598-020-76147-6, PMID: 33168900 PMC7652873

[ref44] Zhao Y. L. Xiang Q. Shi Q. Y. Li S. Y. Tan L. Wang J. T.. (2011). GABAergic excitotoxicity injury of the immature hippocampal pyramidal neurons’ exposure to isoflurane. Anesth. Analg. 113, 1152–1160. doi: 10.1213/ANE.0b013e318230b3fd 21918167

[ref45] Zheng Y. Ren Z. Liu Y. Yan J. Chen C. He Y.. (2024). T cell interactions with microglia in immune-inflammatory processes of ischemic stroke. Neural Regen. Res. 20, 1277–1292. doi: 10.4103/NRR.NRR-D-23-01385 39075894 PMC11624874

[ref46] Zuo Z. (2012). Are volatile anesthetics neuroprotective or neurotoxic? Med. Gas Res. 2:10. doi: 10.1186/2045-9912-2-10, PMID: 22510328 PMC3353836

